# Crystal structure and Hirshfeld surface analysis of 4-(4-chloro­phen­yl)-5-methyl-3-{4-[(2-methyl­phen­yl)meth­oxy]phen­yl}-1,2-oxazole

**DOI:** 10.1107/S2056989021002383

**Published:** 2021-03-05

**Authors:** Abdullah Aydin, Mehmet Akkurt, Sumeyye Turanli, Deniz Lengerli, Erden Banoglu, Nefise Dilek Ozcelik

**Affiliations:** a Department of Mathematics and Science Education, Faculty of Education, Kastamonu University, 37200 Kastamonu, Turkey; b Department of Physics, Faculty of Sciences, Erciyes University, 38039 Kayseri, Turkey; c Department of Pharmaceutical Chemistry, Faculty of Pharmacy, Gazi University, 06330 Ankara, Turkey; d Department of Physics, Faculty of Arts and Sciences, Aksaray University, 68100 Aksaray, Turkey

**Keywords:** crystal structure, vicinal diaryl isoxazole, C—H⋯π inter­actions, Hirshfeld surface analysis

## Abstract

In the title compound, C_24_H_20_ClNO_2_, the mean planes of 4-chloro­phenyl, 2-methyl­phenyl and phenyl­ene rings make dihedral angles of 62.8 (2), 65.1 (3) and 15.1 (2)°, respectively, with the 5-methyl-1,2-oxazole ring. In the crystal, mol­ecules are linked by inter­molecular C—H⋯N, C—H⋯Cl, C—H⋯π contacts and π–π stacking inter­actions between the phenyl­ene groups. Hirshfeld surface analysis indicates that the most important contributions to the crystal packing are from H⋯H (48.7%), H⋯C/C⋯H (22.2%), Cl⋯H/H⋯Cl (8.8%), H⋯O/O⋯H (8.2%) and H⋯N/N⋯H (5.1%) inter­actions.

## Abstract 2

In the crystal, the title mol­ecules are linked by inter­molecular C—H⋯N, C—H⋯Cl, C—H⋯π contacts and π–π stacking inter­actions. A Hirshfeld surface analysis was undertaken to qu­antify the inter­molecular inter­actions.

## Chemical context   

Azoles are five-membered heterocycles that have been widely used as promising scaffolds in designing novel therapeutics, in particular anti­cancer agents (Ahmad *et al.*, 2018 ▸). Among them, isoxazole, a five-membered heterocycle with consecutive nitro­gen and oxygen atoms in the ring, is found to be a key structural component of many commercial drugs or drug candidates in clinical development (Barmade *et al.*, 2016 ▸). Moreover, a number of vicinal diaryl isoxazoles reported in the literature exhibit anti­cancer and COX-2 inhibitory activities, such as luminesbip and valdexocib, respectively (Murumkar & Ghuge, 2018 ▸). One of the critical steps in rational drug design is obtaining knowledge of the structure of the new drug candidates, and single-crystal X-ray diffraction (SCXD) is one of the most powerful methods for gaining this fundamental information, which can be used to guide the drug-design studies in connection with other technologies such as pharmacophore model elaborations, 3D QSAR, docking, and *de novo* design. SCXD has thus become an essential tool for drug development to unambiguously determine the three-dimensional structures of mol­ecules, which eventually paves the way for rapid development of new mol­ecules (Wouters & Ooms, 2001 ▸). Moreover, during the drug-development process, another important issue lies in understanding the crystal packing of the active pharmaceutical ingredient (drug substance) for suitable formulation development. Since most drug mol­ecules comprise solid dosage forms in the crystalline state, it is imperative to truly understand the relationships between the crystal structures and the solid properties of pharmaceutically active substances, which helps the best form of an active pharmaceutical ingredient to be chosen for development into a drug product (Aitipamula & Vangala, 2017 ▸). Based on the above and our continuing inter­est in structural studies and biological applications of diaryl heterocycles (Banoglu *et al.*, 2016 ▸; Çalışkan *et al.*, 2011 ▸; Dündar *et al.*, 2009 ▸; Eren *et al.*, 2010 ▸; Ergun *et al.*, 2010 ▸; Garscha *et al.*, 2016 ▸; Levent *et al.*, 2013 ▸; Pirol *et al.*, 2014 ▸; Ünlü *et al.*, 2007 ▸), we report herein the crystal structure and Hirshfeld surface analysis of the title compound.
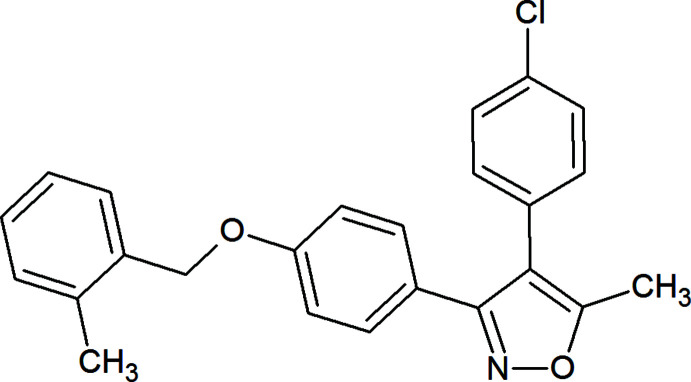


## Structural commentary   

**Figure 1:**
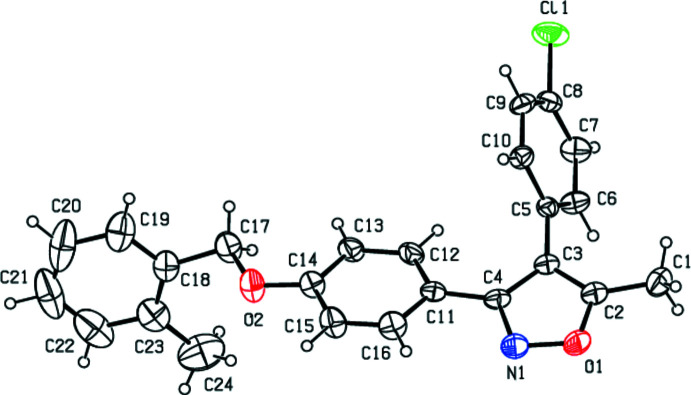
Mol­ecular structure of the title compound with the atom-numbering scheme. Displacement ellipsoids for non-H atoms are drawn at the 30% probability level.

In the mol­ecule of the title compound (Fig. 1 ▸), the mean planes of 4-chloro­phenyl, 2-methyl­phenyl and phenyl­ene rings form dihedral angles of 62.8 (2), 65.1 (3) and 15.1 (2)°, respectively, with respect to the 5-methyl-1,2-oxazole ring. The 4-chloro­phenyl ring makes dihedral angles of 77.4 (3) and 66.38 (19)°, respectively, with the 2-methyl­phenyl and phenyl­ene rings, while the dihedral angle between the 2-methyl­phenyl and phenyl­ene rings is 80.0 (3)°. The C14—O2—C17—C18 torsion angle is 166.7 (4)°. The terminal 2-methyl­phenyl group is involved in intense thermal motion.

## Supra­molecular features   

**Figure 2:**
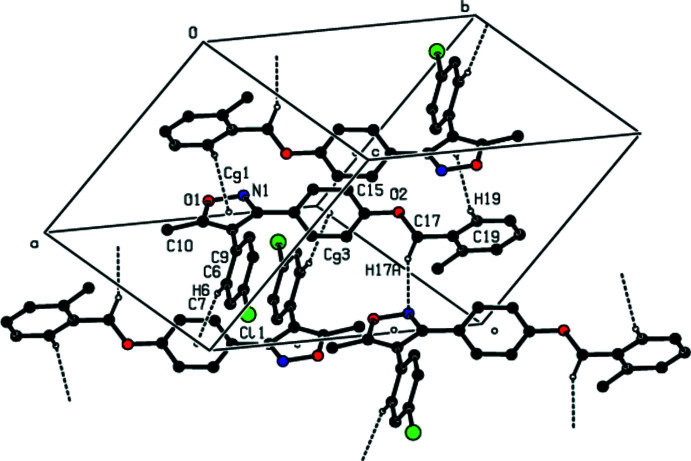
A view of the C—H⋯N and C—H⋯π inter­actions in the unit cell of the title compound. Dashed lines show short inter­molecular contacts.

**Figure 3:**
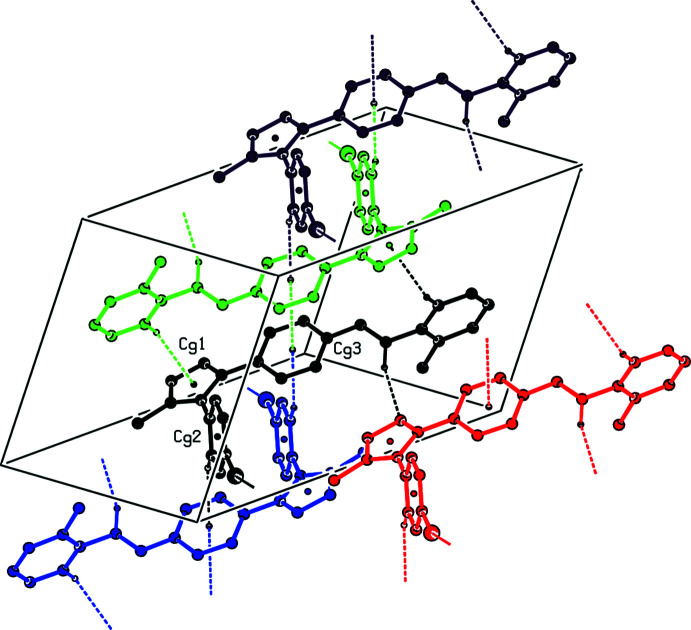
A view of the C—H⋯N and C—H⋯π and π–π inter­actions in the unit cell of the title compound. Dashed lines show short inter­molecular contacts.

In the crystal, mol­ecules are linked by inter­molecular C—H⋯N, C—H⋯Cl and C—H⋯π contacts (Table 1 ▸, Fig. 2 ▸) and π–π inter­actions between inversion-related phenyl­ene rings [inter­centroid separation *Cg*3⋯*Cg*3(1 − *x*, 1 − *y*, 1 − *z*) = 3.958 (2) Å] (Fig. 3 ▸).

## Hirshfeld surface analysis   

**Figure 4:**
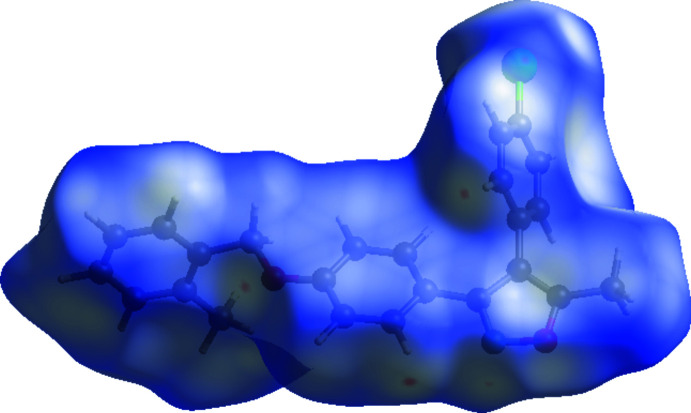
The Hirshfeld surface of the title compound mapped with *d*_norm_.

Hirshfeld surface analysis (Hirshfeld, 1977 ▸; Spackman & Jayatilaka, 2009 ▸) of the title compound was carried out to investigate the location of atoms with potential to form hydrogen bonds and other inter­molecular contacts, and the qu­anti­tative ratio of these inter­actions. *Crystal Explorer17.5* (Turner *et al.*, 2017 ▸) was used to generate the Hirshfeld surfaces and two-dimensional fingerprint plots (Rohl *et al.*, 2008 ▸). The Hirshfeld surfaces were generated using a standard (high) surface resolution with the three-dimensional *d*_norm_ surfaces mapped over a fixed colour scale of −0.0800 (red) to 1.5787 Å (blue) (Fig. 4 ▸).

The red points, which represent closer contacts and negative *d*_norm_ values on the surface, correspond to the C—H⋯N (C17—H17*A*⋯N1), C—H⋯Cl (C8—Cl1⋯H1*C*—C1) and C—H⋯π (C6—H6⋯phenyl­ene) inter­actions (Table 2 ▸). Except for the red spots, the overall surface mapped over *d*_norm_ is white and blue, indicating that the distances between the contact atoms in inter­molecular contacts are nearly the same as the sum of their van der Waals radii or longer.

**Figure 5:**
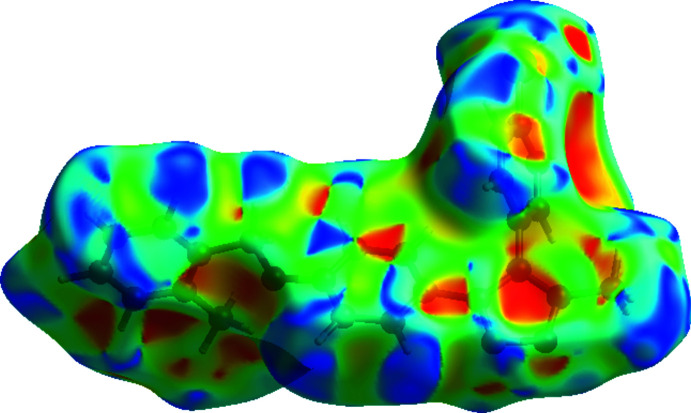
Hirshfeld surface of the title compound plotted over shape-index.

The shape-index of the Hirshfeld surface is a tool for visualizing the π–π stacking by the presence of adjacent red and blue triangles; if there are no such triangles, then there are no π–π inter­actions. The plot of the Hirshfeld surface mapped over shape-index clearly suggests that there are π–π inter­actions in the title compound (Fig. 5 ▸).

**Figure 6:**
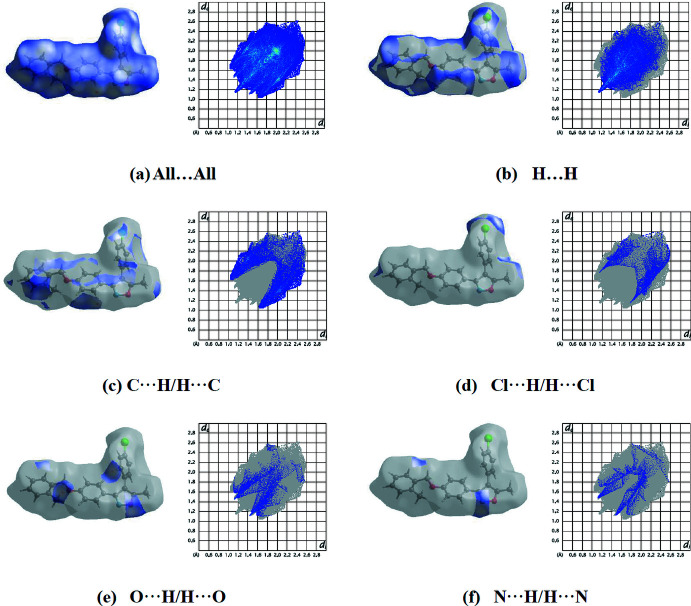
The total two-dimensional fingerprint plot (*a*) and the relative contributions of various inter­actions to the Hirshfeld surface: (*b*) H⋯H, (*c*) H⋯C/C⋯H, (*d*) Cl⋯H/H⋯Cl, (*e*) H⋯O/O⋯H and (*f*) H⋯N/N⋯H.

Fig. 6 ▸(*a*) shows the total two-dimensional fingerprint plot providing information on the major and minor percentage contributions of the inter­atomic contacts to the Hirshfeld surface of the title compound. The blue colour refers to the frequency of occurrence of the (*d*_i_, *d*_e_) pair and the grey colour is the outline of the full fingerprint (Zaini *et al.*, 2019 ▸). The fingerprint plots (Fig. 6 ▸ *b*) show that the H⋯H contacts clearly make the most significant contribution to the Hirshfeld surface (48.7%). The H⋯C/C⋯H, Cl⋯H/H⋯Cl, H⋯O/O⋯H and H⋯N/N⋯H contacts contribute 22.2, 8.8, 8.2 and 5.1%, respectively (Fig. 6 ▸ *c–f*). The remaining weaker contacts are listed in Table 3 ▸.

The large number of H⋯H, H⋯C/C⋯H, Cl⋯H/H⋯Cl, H⋯O/O⋯H and H⋯N/N⋯H inter­actions suggest that van der Waals inter­actions play the major roles in the crystal packing (Hathwar *et al.*, 2015 ▸).

## Database survey   

The closest related 1,2-oxazole compounds containing a halogen atom, but with different substituents at the aromatic rings are: ethyl 3-(4-chloro­phen­yl)-5-[(*E*)-2- (di­methyl­amino)­ethen­yl]-1,2-oxazole-4- carboxyl­ate [(I); Efimov *et al.*, 2015 ▸], *N*-(2,4-di­fluoro­phen­yl)-5-methyl-1,2-oxazole-4-carboxamide hemihydrate [(II); Yu *et al.*, 2012 ▸] and *N*-(2,6-di­chloro­phen­yl)-5-methyl-1,2-oxazole-4-carboxamide monohydrate [(III); Wang *et al.*, 2011 ▸].

In compound (I), the asymmetric unit contains two mol­ecules, *A* and *B*, with different conformations. In mol­ecule *A*, the C=O group of the ester points away from the benzene ring [C—C—C=O = −170.8 (3)°], whereas in mol­ecule *B*, it points back towards the benzene ring [C—C—C=O = 17.9 (4)°]. The dihedral angles between the oxazole and benzene rings are also somewhat different [46.26 (13) and 41.59 (13)° for mol­ecules *A* and *B*, respectively]. Each mol­ecule features an intra­molecular C—H⋯O inter­action, which closes an *S*(6) ring. In the crystal, the *B* mol­ecules are linked into *C*(12) chains along the *c*-axis direction by weak C—H⋯Cl inter­actions. In the crystal of (II), the components are linked by O—H⋯N and N—H⋯O hydrogen bonds, where the water mol­ecule acts as both an H-atom donor and an acceptor, into a tape along the *a-*axis direction with an 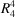
(16) graph-set motif. The water mol­ecule is located on a twofold rotation axis. In (III), the dihedral angle between the benzene and isoxazole rings is 59.10 (7)°. In the crystal, the components are linked by N—H⋯O and O—H⋯O hydrogen bonds into a three-dimensional network. The crystal structure is further stabilized by π-stacking inter­actions [inter­centroid distance = 3.804 (2) Å].

## Synthesis and crystallization   

Step 1: To a solution of *N*-hy­droxy-4-[(2-methyl­benz­yl)­oxy]benzimidoyl chloride (275 mg, 1 mmol) in diethyl ether (6 ml) was added Et_3_N (139.4 µL, 1 mmol). The resulting mixture was stirred for 2 h in an ice bath, and the precipitate formed was filtered off. The filtrate was evaporated under vacuum to obtain the aryl­nitriloxide inter­mediate.

Step 2: To a solution of NaH (60% in mineral oil, 64 mg, 1.6 mmol) in dry THF (4 ml), 4-chloro­phenyl­acetone (168,6 mg, 1.0 mmol) was added dropwise, and stirred for 1 h under a nitro­gen atmosphere in an ice bath. At the end of the period, the aryl­nitriloxide inter­mediate was dissolved in dry THF (4 ml), and was added to the reaction mixture, then stirred at room temperature overnight. Upon completion of the reaction, aqueous ammonium chloride solution was added, and the product was extracted with EtOAc (2 × 50 mL). The combined organic extracts were dried over anhydrous Na_2_SO_4_, filtered and evaporated to dryness. The crude product was purified by automated-flash chromatography on silica gel (12 g) eluting with a gradient of 0 to 40% EtOAc in hexane. The obtained pure product was recrystallized from methanol. Crystals for structural study were obtained by slow cooling of the solution, yield 77%, m.p. 387.2–388.6 K.

^1^H NMR (400 MHz, CDCl_3_): δ 2.29 (3H, *s*), 2.39 (3H, *s*), 5.07 (2H, *s*), 7.05 (2H, *d*, *J* = 8.4 Hz), 7.15–7.25 (5H, *m*), 7.27 (2H, *d*, *J* = 8.8 Hz), 7.38 (1H, *d*, *J* = 7.6 Hz), 7.47 (2H, *d*, *J* = 8.4 Hz).

^13^C NMR (100 MHz, CDCl_3_): δ 11.21, 18.42, 67.98, 113.96, 114.97, 120.84, 125.77, 128.15, 128.59, 128.86, 129.43, 130.12, 131.44, 132.57, 134.58, 136.64, 159.49, 160.09, 166.93. HRMS (*m*/*z*): [*M* + H]^+^ calculated for C_24_H_21_ClNO_2_: 390.1261; found: 390.1263.

## Refinement   

Crystal data, data collection and structure refinement details are summarized in Table 4 ▸. H atoms were positioned geometrically (C—H = 0.93–0.97 Å) and refined as riding with *U*_iso_(H) = 1.2*U*_eq_(C) or 1.5*U*_eq_(C-meth­yl). In the final refinement, three outliers (1 11 9, 2 16 7, 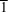 19 6) were omitted.

## Supplementary Material

Crystal structure: contains datablock(s) I. DOI: 10.1107/S2056989021002383/yk2147sup1.cif

Structure factors: contains datablock(s) I. DOI: 10.1107/S2056989021002383/yk2147Isup2.hkl

Supporting information file. DOI: 10.1107/S2056989021002383/yk2147Isup3.cml

CCDC reference: 2067449

Additional supporting information:  crystallographic information; 3D view; checkCIF report

## Figures and Tables

**Table 1:** Intermolecular contacts (Å, °) *Cg*1, *Cg*2 and *Cg*3 are the centroids of the O1/N1/C2–C4, C5–C10 and C11–C16 rings, respectively.

*D*—H⋯*A*	*D*—H	H⋯*A*	*D*⋯*A*	*D*—H⋯*A*
C17—H17*A*⋯N1^i^	0.97	2.68	3.395 (6)	131
C6—H6⋯*Cg*3^ii^	0.93	2.86	3.747 (5)	159
C19—H19⋯*Cg*1^iii^	0.93	2.77	3.614 (6)	151
C8—Cl1⋯*Cg*2^iv^	1.75 (1)	3.37 (1)	5.034 (4)	159 (1)

Symmetry codes: (i) x, y, z+1; (ii) -x+2, -y+1, -z+1; (iii) -x+1, -y+1, -z+1; (iv) x, -y-{\script{1\over 2}}, z-{\script{1\over 2}}.

**Table 2:** Summary of selected van der Waals contacts (Å) involving H atoms in the title compound

Contact	Distance	Symmetry operation
Cl1⋯H1*C*	3.04	*x*, {1\over 2} − *y*, {1\over 2} + *z*
N1⋯H17*A*	2.68	*x*, *y*, −1 + *z*
H1*A*⋯O1	2.74	2 − *x*, 1 − *y*, −*z*
H10⋯O2	2.72	1 − *x*, 1 − *y*, 1 − *z*
H6⋯C11	2.80	2 − *x*, 1 − *y*, 1 − *z*
H9⋯C21	2.94	1 − *x*, −{1\over 2} + *y*, {3\over 2} − *z*
H20⋯C9	3.05	1 − *x*, 1 − *y*, 2 − *z*
H22⋯H24*C*	2.48	*x*, {1\over 2} − *y*, − {1\over 2} + *z*

**Table 3:** Percentage contributions of inter­atomic contacts to the Hirshfeld surface of the title compound

Contact	Percentage contribution
H⋯H	48.7
H⋯C/C⋯H	22.2
Cl⋯H/H⋯Cl	8.8
H⋯O/O⋯H	8.2
H⋯N/N⋯H	5.1
Cl⋯C/C⋯Cl	3.9
C⋯C	2.1
C⋯N/N⋯C	0.4
O⋯O	0.4
C⋯O/O⋯C	0.2

**Table 4:** Experimental details

Crystal data	
Chemical formula	C_24_H_20_ClNO_2_
*M*_r_	389.86
Crystal system, space group	Monoclinic, *P*2_1_/*c*
Temperature (K)	296
*a*, *b*, *c* (Å)	10.5733 (10), 22.848 (2), 8.7151 (9)
β (°)	101.477 (4)
*V* (Å^3^)	2063.3 (4)
*Z*	4
Radiation type	Mo *K*α
μ (mm^−1^)	0.20
Crystal size (mm)	0.17 × 0.13 × 0.11

Data collection	
Diffractometer	Bruker SMART BREEZE CCD
Absorption correction	Multi-scan (*SADABS*; Bruker, 2007 ▸)
*T*_min_, *T*_max_	0.598, 0.745
No. of measured, independent and observed [*I* > 2σ(*I*)] reflections	45630, 3840, 3030
*R*_int_	0.064
(sin θ/λ)_max_ (Å^−1^)	0.606

Refinement	
*R*[*F* ^2^ > 2σ(*F* ^2^)], *wR*(*F* ^2^), *S*	0.099, 0.187, 1.25
No. of reflections	3840
No. of parameters	255
H-atom treatment	H-atom parameters constrained
Δρ_max_, Δρ_min_ (e Å^−3^)	0.29, −0.33

Computer programs: *APEX2* and *SAINT* (Bruker, 2007 ▸), *SHELXT2014/4* (Sheldrick, 2015*a* ▸), *SHELXL2018/3* (Sheldrick, 2015*b* ▸), *ORTEP-3 for Windows* and *WinGX* (Farrugia, 2012 ▸) and *PLATON* (Spek, 2020 ▸).

## supplementary crystallographic information

### Crystal data

**Table:**

C_24_H_20_ClNO_2_	*F*(000) = 816
*M**_r_* = 389.86	*D*_x_ = 1.255 Mg m^−^^3^
Monoclinic, *P*2_1_/*c*	Mo *K*α radiation, λ = 0.71073 Å
*a* = 10.5733 (10) Å	Cell parameters from 9984 reflections
*b* = 22.848 (2) Å	θ = 2.9–26.1°
*c* = 8.7151 (9) Å	µ = 0.20 mm^−^^1^
β = 101.477 (4)°	*T* = 296 K
*V* = 2063.3 (4) Å^3^	Block, colourless
*Z* = 4	0.17 × 0.13 × 0.11 mm

### Data collection

**Table:**

Bruker SMART BREEZE CCD diffractometer	3030 reflections with *I* > 2σ(*I*)
φ and ω scans	*R*_int_ = 0.064
Absorption correction: multi-scan (SADABS; Bruker, 2007)	θ_max_ = 25.5°, θ_min_ = 2.9°
*T*_min_ = 0.598, *T*_max_ = 0.745	*h* = −12→12
45630 measured reflections	*k* = −27→27
3840 independent reflections	*l* = −10→10

### Refinement

**Table:**

Refinement on *F*^2^	0 restraints
Least-squares matrix: full	Hydrogen site location: inferred from neighbouring sites
*R*[*F*^2^ > 2σ(*F*^2^)] = 0.099	H-atom parameters constrained
*wR*(*F*^2^) = 0.187	*w* = 1/[σ^2^(*F*_o_^2^) + (0.0303*P*)^2^ + 3.1816*P*] where *P* = (*F*_o_^2^ + 2*F*_c_^2^)/3
*S* = 1.25	(Δ/σ)_max_ < 0.001
3840 reflections	Δρ_max_ = 0.29 e Å^−^^3^
255 parameters	Δρ_min_ = −0.33 e Å^−^^3^

### Special details

**Table:**

Geometry. All esds (except the esd in the dihedral angle between two l.s. planes) are estimated using the full covariance matrix. The cell esds are taken into account individually in the estimation of esds in distances, angles and torsion angles; correlations between esds in cell parameters are only used when they are defined by crystal symmetry. An approximate (isotropic) treatment of cell esds is used for estimating esds involving l.s. planes.

### Fractional atomic coordinates and isotropic or equivalent isotropic displacement parameters (Å^2^)

**Table:**

	*x*	*y*	*z*	*U*_iso_*/*U*_eq_	
C1	0.9916 (5)	0.3972 (2)	0.0798 (6)	0.0786 (15)	
H1A	1.073792	0.414990	0.079720	0.118*	
H1B	0.949677	0.388351	−0.025762	0.118*	
H1C	1.003929	0.361730	0.140061	0.118*	
C2	0.9100 (4)	0.43821 (19)	0.1503 (5)	0.0575 (11)	
C3	0.8681 (4)	0.43905 (16)	0.2861 (4)	0.0445 (9)	
C4	0.7911 (4)	0.49060 (16)	0.2788 (4)	0.0477 (9)	
C5	0.8951 (3)	0.39390 (15)	0.4106 (4)	0.0399 (8)	
C6	1.0198 (4)	0.38314 (18)	0.4902 (5)	0.0559 (11)	
H6	1.087573	0.404533	0.464626	0.067*	
C7	1.0458 (4)	0.34153 (19)	0.6061 (5)	0.0601 (11)	
H7	1.129976	0.334858	0.658977	0.072*	
C8	0.9461 (4)	0.31025 (16)	0.6423 (4)	0.0505 (10)	
C9	0.8221 (4)	0.31897 (17)	0.5654 (4)	0.0513 (10)	
H9	0.755403	0.296826	0.590784	0.062*	
C10	0.7965 (4)	0.36096 (17)	0.4494 (4)	0.0474 (9)	
H10	0.712000	0.367117	0.396865	0.057*	
C11	0.7223 (4)	0.51653 (15)	0.3940 (4)	0.0434 (9)	
C12	0.7465 (4)	0.50001 (17)	0.5498 (5)	0.0534 (10)	
H12	0.804645	0.469849	0.582691	0.064*	
C13	0.6866 (4)	0.52715 (17)	0.6581 (5)	0.0560 (11)	
H13	0.703823	0.515063	0.762061	0.067*	
C14	0.6014 (4)	0.57204 (18)	0.6108 (4)	0.0511 (10)	
C15	0.5757 (4)	0.5890 (2)	0.4563 (5)	0.0638 (12)	
H15	0.517749	0.619299	0.423717	0.077*	
C16	0.6351 (4)	0.5615 (2)	0.3503 (5)	0.0604 (11)	
H16	0.616425	0.573436	0.246158	0.072*	
C17	0.5646 (5)	0.5876 (2)	0.8714 (5)	0.0726 (14)	
H17A	0.656655	0.583009	0.909755	0.087*	
H17B	0.522468	0.550907	0.885738	0.087*	
C18	0.5135 (4)	0.6354 (2)	0.9591 (4)	0.0599 (12)	
C19	0.4002 (5)	0.6258 (3)	1.0119 (6)	0.0900 (17)	
H19	0.356626	0.590511	0.988715	0.108*	
C20	0.3513 (7)	0.6669 (5)	1.0973 (8)	0.130 (3)	
H20	0.275977	0.659313	1.133673	0.156*	
C21	0.4116 (11)	0.7181 (4)	1.1287 (9)	0.136 (4)	
H21	0.377369	0.746428	1.185453	0.163*	
C22	0.5229 (9)	0.7290 (3)	1.0780 (8)	0.117 (3)	
H22	0.563766	0.764957	1.100443	0.140*	
C23	0.5775 (6)	0.6873 (3)	0.9926 (6)	0.0797 (15)	
C24	0.7025 (7)	0.6991 (3)	0.9434 (8)	0.134 (3)	
H24A	0.769778	0.676652	1.007418	0.200*	
H24B	0.722625	0.739980	0.955410	0.200*	
H24C	0.695494	0.688090	0.835677	0.200*	
Cl1	0.97763 (16)	0.25691 (6)	0.78820 (15)	0.0889 (5)	
N1	0.7874 (4)	0.51849 (16)	0.1458 (4)	0.0666 (10)	
O1	0.8631 (3)	0.48497 (14)	0.0621 (3)	0.0709 (9)	
O2	0.5395 (3)	0.60330 (14)	0.7082 (3)	0.0668 (9)	

### Atomic displacement parameters (Å^2^)

**Table:**

	*U*^11^	*U*^22^	*U*^33^	*U*^12^	*U*^13^	*U*^23^
C1	0.092 (4)	0.084 (4)	0.071 (3)	0.014 (3)	0.042 (3)	0.013 (3)
C2	0.066 (3)	0.054 (2)	0.055 (2)	0.000 (2)	0.019 (2)	0.012 (2)
C3	0.049 (2)	0.043 (2)	0.041 (2)	−0.0046 (17)	0.0083 (17)	0.0078 (17)
C4	0.058 (2)	0.045 (2)	0.040 (2)	−0.0057 (18)	0.0081 (17)	0.0113 (17)
C5	0.049 (2)	0.0339 (18)	0.0374 (19)	−0.0043 (16)	0.0101 (16)	−0.0036 (15)
C6	0.051 (2)	0.058 (3)	0.057 (2)	−0.014 (2)	0.0059 (19)	0.010 (2)
C7	0.056 (3)	0.062 (3)	0.056 (3)	−0.002 (2)	−0.005 (2)	0.010 (2)
C8	0.078 (3)	0.034 (2)	0.040 (2)	0.001 (2)	0.014 (2)	0.0018 (16)
C9	0.059 (3)	0.050 (2)	0.049 (2)	−0.018 (2)	0.020 (2)	0.0019 (19)
C10	0.042 (2)	0.052 (2)	0.049 (2)	−0.0019 (18)	0.0104 (17)	0.0035 (18)
C11	0.049 (2)	0.038 (2)	0.040 (2)	−0.0029 (17)	0.0022 (16)	0.0068 (16)
C12	0.068 (3)	0.041 (2)	0.051 (2)	0.0125 (19)	0.011 (2)	0.0115 (18)
C13	0.078 (3)	0.049 (2)	0.040 (2)	0.012 (2)	0.009 (2)	0.0093 (18)
C14	0.051 (2)	0.056 (2)	0.043 (2)	0.0058 (19)	−0.0006 (18)	−0.0016 (19)
C15	0.064 (3)	0.072 (3)	0.048 (2)	0.028 (2)	−0.006 (2)	0.006 (2)
C16	0.071 (3)	0.072 (3)	0.034 (2)	0.013 (2)	−0.0016 (19)	0.009 (2)
C17	0.092 (4)	0.073 (3)	0.052 (3)	0.026 (3)	0.012 (2)	0.003 (2)
C18	0.058 (3)	0.080 (3)	0.037 (2)	0.022 (2)	−0.0031 (19)	0.006 (2)
C19	0.074 (3)	0.134 (5)	0.059 (3)	0.015 (3)	0.004 (3)	−0.013 (3)
C20	0.093 (5)	0.231 (10)	0.067 (4)	0.062 (6)	0.018 (4)	−0.017 (6)
C21	0.174 (9)	0.150 (8)	0.073 (5)	0.103 (8)	−0.004 (5)	−0.019 (5)
C22	0.172 (8)	0.086 (4)	0.071 (4)	0.023 (5)	−0.024 (5)	−0.013 (3)
C23	0.088 (4)	0.085 (4)	0.055 (3)	0.011 (3)	−0.011 (3)	0.000 (3)
C24	0.116 (6)	0.157 (7)	0.118 (6)	−0.049 (5)	0.000 (4)	0.011 (5)
Cl1	0.1332 (12)	0.0644 (8)	0.0662 (8)	0.0031 (8)	0.0132 (8)	0.0303 (6)
N1	0.086 (3)	0.061 (2)	0.057 (2)	0.015 (2)	0.024 (2)	0.0205 (18)
O1	0.095 (2)	0.072 (2)	0.0546 (18)	0.0134 (18)	0.0346 (17)	0.0237 (16)
O2	0.073 (2)	0.083 (2)	0.0402 (16)	0.0342 (17)	0.0011 (14)	0.0025 (15)

### Geometric parameters (Å, º)

**Table:**

C1—C2	1.487 (6)	C13—H13	0.9300
C1—H1A	0.9600	C14—O2	1.371 (5)
C1—H1B	0.9600	C14—C15	1.376 (5)
C1—H1C	0.9600	C15—C16	1.369 (6)
C2—C3	1.344 (5)	C15—H15	0.9300
C2—O1	1.351 (5)	C16—H16	0.9300
C3—C4	1.426 (5)	C17—O2	1.439 (5)
C3—C5	1.483 (5)	C17—C18	1.494 (6)
C4—N1	1.317 (5)	C17—H17A	0.9700
C4—C11	1.476 (5)	C17—H17B	0.9700
C5—C10	1.381 (5)	C18—C23	1.368 (7)
C5—C6	1.384 (5)	C18—C19	1.383 (7)
C6—C7	1.375 (5)	C19—C20	1.364 (9)
C6—H6	0.9300	C19—H19	0.9300
C7—C8	1.361 (6)	C20—C21	1.334 (12)
C7—H7	0.9300	C20—H20	0.9300
C8—C9	1.363 (6)	C21—C22	1.361 (11)
C8—Cl1	1.745 (4)	C21—H21	0.9300
C9—C10	1.381 (5)	C22—C23	1.402 (9)
C9—H9	0.9300	C22—H22	0.9300
C10—H10	0.9300	C23—C24	1.493 (8)
C11—C16	1.383 (5)	C24—H24A	0.9600
C11—C12	1.383 (5)	C24—H24B	0.9600
C12—C13	1.384 (5)	C24—H24C	0.9600
C12—H12	0.9300	N1—O1	1.411 (4)
C13—C14	1.373 (5)		
			
C2—C1—H1A	109.5	O2—C14—C13	124.7 (3)
C2—C1—H1B	109.5	O2—C14—C15	115.7 (3)
H1A—C1—H1B	109.5	C13—C14—C15	119.6 (4)
C2—C1—H1C	109.5	C16—C15—C14	120.2 (4)
H1A—C1—H1C	109.5	C16—C15—H15	119.9
H1B—C1—H1C	109.5	C14—C15—H15	119.9
C3—C2—O1	110.0 (4)	C15—C16—C11	121.7 (4)
C3—C2—C1	133.8 (4)	C15—C16—H16	119.1
O1—C2—C1	116.2 (4)	C11—C16—H16	119.1
C2—C3—C4	104.9 (3)	O2—C17—C18	108.0 (3)
C2—C3—C5	125.8 (4)	O2—C17—H17A	110.1
C4—C3—C5	129.2 (3)	C18—C17—H17A	110.1
N1—C4—C3	110.8 (3)	O2—C17—H17B	110.1
N1—C4—C11	118.2 (3)	C18—C17—H17B	110.1
C3—C4—C11	131.0 (3)	H17A—C17—H17B	108.4
C10—C5—C6	118.1 (3)	C23—C18—C19	119.4 (5)
C10—C5—C3	120.9 (3)	C23—C18—C17	121.9 (5)
C6—C5—C3	121.0 (3)	C19—C18—C17	118.6 (5)
C7—C6—C5	121.4 (4)	C20—C19—C18	121.3 (7)
C7—C6—H6	119.3	C20—C19—H19	119.4
C5—C6—H6	119.3	C18—C19—H19	119.4
C8—C7—C6	118.9 (4)	C21—C20—C19	119.9 (8)
C8—C7—H7	120.6	C21—C20—H20	120.0
C6—C7—H7	120.6	C19—C20—H20	120.0
C7—C8—C9	121.5 (4)	C20—C21—C22	120.2 (8)
C7—C8—Cl1	119.4 (3)	C20—C21—H21	119.9
C9—C8—Cl1	119.1 (3)	C22—C21—H21	119.9
C8—C9—C10	119.4 (3)	C21—C22—C23	121.5 (8)
C8—C9—H9	120.3	C21—C22—H22	119.3
C10—C9—H9	120.3	C23—C22—H22	119.3
C9—C10—C5	120.7 (4)	C18—C23—C22	117.7 (6)
C9—C10—H10	119.7	C18—C23—C24	121.5 (6)
C5—C10—H10	119.7	C22—C23—C24	120.8 (7)
C16—C11—C12	117.1 (4)	C23—C24—H24A	109.5
C16—C11—C4	120.2 (3)	C23—C24—H24B	109.5
C12—C11—C4	122.6 (3)	H24A—C24—H24B	109.5
C11—C12—C13	121.8 (4)	C23—C24—H24C	109.5
C11—C12—H12	119.1	H24A—C24—H24C	109.5
C13—C12—H12	119.1	H24B—C24—H24C	109.5
C14—C13—C12	119.5 (4)	C4—N1—O1	105.8 (3)
C14—C13—H13	120.3	C2—O1—N1	108.5 (3)
C12—C13—H13	120.3	C14—O2—C17	117.7 (3)
			
O1—C2—C3—C4	−0.5 (5)	C12—C13—C14—O2	−177.9 (4)
C1—C2—C3—C4	−179.0 (5)	C12—C13—C14—C15	0.7 (7)
O1—C2—C3—C5	177.6 (3)	O2—C14—C15—C16	178.4 (4)
C1—C2—C3—C5	−0.9 (8)	C13—C14—C15—C16	−0.3 (7)
C2—C3—C4—N1	0.2 (5)	C14—C15—C16—C11	−0.3 (7)
C5—C3—C4—N1	−177.8 (4)	C12—C11—C16—C15	0.5 (6)
C2—C3—C4—C11	−177.8 (4)	C4—C11—C16—C15	−175.9 (4)
C5—C3—C4—C11	4.2 (7)	O2—C17—C18—C23	−77.0 (5)
C2—C3—C5—C10	−115.9 (5)	O2—C17—C18—C19	105.2 (5)
C4—C3—C5—C10	61.7 (5)	C23—C18—C19—C20	−0.3 (8)
C2—C3—C5—C6	63.4 (6)	C17—C18—C19—C20	177.7 (5)
C4—C3—C5—C6	−119.0 (5)	C18—C19—C20—C21	1.3 (10)
C10—C5—C6—C7	−0.9 (6)	C19—C20—C21—C22	−1.1 (12)
C3—C5—C6—C7	179.8 (4)	C20—C21—C22—C23	−0.2 (11)
C5—C6—C7—C8	0.3 (7)	C19—C18—C23—C22	−1.0 (7)
C6—C7—C8—C9	0.6 (6)	C17—C18—C23—C22	−178.8 (4)
C6—C7—C8—Cl1	179.5 (3)	C19—C18—C23—C24	177.7 (5)
C7—C8—C9—C10	−0.8 (6)	C17—C18—C23—C24	−0.1 (7)
Cl1—C8—C9—C10	−179.8 (3)	C21—C22—C23—C18	1.2 (9)
C8—C9—C10—C5	0.2 (6)	C21—C22—C23—C24	−177.5 (6)
C6—C5—C10—C9	0.6 (6)	C3—C4—N1—O1	0.2 (5)
C3—C5—C10—C9	180.0 (3)	C11—C4—N1—O1	178.4 (3)
N1—C4—C11—C16	13.4 (6)	C3—C2—O1—N1	0.6 (5)
C3—C4—C11—C16	−168.7 (4)	C1—C2—O1—N1	179.4 (4)
N1—C4—C11—C12	−162.7 (4)	C4—N1—O1—C2	−0.5 (5)
C3—C4—C11—C12	15.1 (6)	C13—C14—O2—C17	−0.5 (6)
C16—C11—C12—C13	0.0 (6)	C15—C14—O2—C17	−179.2 (4)
C4—C11—C12—C13	176.2 (4)	C18—C17—O2—C14	166.7 (4)
C11—C12—C13—C14	−0.5 (7)		

### Hydrogen-bond geometry (Å, º)

*Cg*1, *Cg*2 and *Cg*3 are the centroids of the O1/N1/C2–C4, C5–C10 and C11–C16 rings, respectively.

**Table:**

*D*—H···*A*	*D*—H	H···*A*	*D*···*A*	*D*—H···*A*
C17—H17*A*···N1^i^	0.97	2.68	3.395 (6)	131
C6—H6···*Cg*3^ii^	0.93	2.86	3.747 (5)	159
C19—H19···*Cg*1^iii^	0.93	2.77	3.614 (6)	151
C8—Cl1···*Cg*2^iv^	1.75 (1)	3.37 (1)	5.034 (4)	159 (1)

Symmetry codes: (i) *x*, *y*, *z*+1; (ii) −*x*+2, −*y*+1, −*z*+1; (iii) −*x*+1, −*y*+1, −*z*+1; (iv) *x*, −*y*−1/2, *z*−1/2.
